# What Are the Effects of a Mediterranean Diet on Allergies and Asthma in Children?

**DOI:** 10.3389/fped.2017.00072

**Published:** 2017-04-21

**Authors:** Jose A. Castro-Rodriguez, Luis Garcia-Marcos

**Affiliations:** ^1^Department of Pediatric Pulmonology, Division of Pediatrics, School of Medicine, Pontificia Universidad Católica de Chile, Santiago, Chile; ^2^Pediatric Allergy and Pulmonology Units, “Virgen de la Arrixaca” University Children’s Hospital, IMIB-Arrixaca Bio-health Research Institute, University of Murcia, Murcia, Spain

**Keywords:** allergic rhinitis, asthma, children, dermatitis, Mediterranean diet, review, wheezing

## Abstract

This review updates the relationship between the adherence to Mediterranean diet (MedDiet) assessed by questionnaire and asthma, allergic rhinitis, or atopic eczema in childhood. It deals with the effect of MedDiet in children on asthma/wheeze, allergic rhinitis, and atopic dermatitis/eczema, and also with the effect of MedDiet consumption by the mother during pregnancy on the inception of asthma/wheeze and allergic diseases in the offspring. Adherence to MedDiet by children themselves seems to have a protective effect on asthma/wheezing symptoms after adjustment for confounders, although the effect is doubtful on lung function and bronchial hyperresponsiveness. By contrast, the vast majority of the studies showed no significant effect of MedDiet on preventing atopic eczema, rhinitis, or atopy. Finally, studies on adherence to MedDiet by the mother during pregnancy showed some protective effect on asthma/wheeze symptoms in the offspring only during the first year of life, but not afterward. Very few studies have shown a protective effect on wheezing, current sneeze, and atopy, and none on eczema. Randomized control trials on the effect of the adherence to MedDiet to prevent (by maternal consumption during pregnancy) or improve (by child consumption) the clinical control of asthma/wheezing, allergic rhinitis, or atopic dermatitis are needed.

## Introduction

Diseases such as asthma, allergic rhinitis, or atopic eczema have increased in the last decades, and the highest incidence seems to occur among children (1). One of the explanations of that increase from the environmental pint of view relates to changes in diet (2). The main components of the modern diet are foods which have been highly processed, modified, stored, and transported over great distances. Furthermore, some reports show a trend to consume higher amounts of saturated fats, such as burgers, and sugar such as soft drinks (3). By contrast, the traditional diet is produced and marketed locally and is eaten shortly after harvesting (4).

One of these traditional diets is the Mediterranean diet (MedDiet). The term MedDiet refers to dietary patterns in the Mediterranean region. MedDiet is characterized by a high intake of fruits and vegetables, bread and whole grain cereals, legumes, and nuts; low-to-moderate consumption of dairy products and eggs; and limited amounts of meat and poultry. This diet is low in saturated fatty acids and rich in antioxidants, carbohydrates, and fiber. It also has a high content of monounsaturated fatty acids and *n* − 3 polyunsaturated fatty acids (PUFAs), mainly derived from olive oil (from fish in some areas) (5).

In the last decade, several epidemiological studies have reported a protective effect of MedDiet on asthma, rhinitis, and atopic eczema, while others did not find any protective effect of this diet on those conditions. Therefore, before nutritional therapy could be included in guidelines devoted to preventing the inception of asthma and allergic diseases in childhood, or to improving the clinical course of those diseases, it is necessary to have a complete view of the current evidence from epidemiological studies. Then, it is time to perform randomized clinical trials (RCTs).

The aim of the present review is to update the evidence on whether the adherence to MedDiet has some effect on asthma, allergic rhinitis, and atopic eczema in childhood.

## What is the Evidence so Far?

We conducted an electronic literature search using Medline/PubMed and EMBASE in September 2016 and extended back to 1960. The terms used to search both in titles and in abstracts were “(((Mediterranean Diet)) AND ((asthma OR wheezing)) OR ((allergic rhinitis)) OR ((atopic dermatitis OR atopic eczema)) AND ((children))).” Only original studies with human subjects were included. No restriction was made for publication language or publishing status. Also, cross-referencing from the articles found was used to complete the search.

A total of 46 studies were retrieved electronically. Among those, 24 studies were excluded for the following reasons: reviews (*n* = 9), editorials (*n* = 6), systematic reviews (*n* = 4), no inclusion of MedDiet (*n* = 1), adult population (*n* = 2), different outcome (*n* = 1), and clinical trial design (*n* = 1). Therefore, 22 original studies were included in the present review. All were observational studies (cross-sectional, cohort, or case–control studies) and assessed the adherence to MedDiet by dietary information collected using food frequency questionnaires and scoring MedDiet by means of different scores.

### Effect of MedDiet Consumption by Children on Asthma/Wheeze

Fifteen studies were retrieved (Table 1) on this topic. We previously reported (6) eight of these studies (7–14) in a systematic review with a meta-analysis. In that review on 39,804 children, we found a negative association of “current wheezing” with the highest tertile of MedDiet score adherence (OR 0.85 [0.75–0.98]; *p* = 0.02). This result was driven by Mediterranean centers (0.79 [0.66–0.94], *p* = 0.009). A similar figure was found for “current severe wheeze” (OR 0.82 [0.55–1.22], *p* = 0.330 for all centers; and OR: 0.66 [0.48–0.90], *p* = 0.008 for Mediterranean centers). Mediterranean centers were centers <100 km from the Mediterranean coast.

**Table 1:** **Summary of studies reporting the association between adherence to MedDiet and asthma/wheezing, allergic rhinitis, and dermatitis/eczema in children**.

Reference	Country	Sample	Study design	MedDiet score	Outcomes	Primary results: effect of MedDiet	Adjusted confounders
Sanchez-Solis et al. (7)	Spain	683 (6–8 years)	C-S	Mod.Psal 1^b^	Clinical significant asthma	adjOR = 0.78 [0.61–0.97]	Percent body fat

Chatzi et al. (9)	Greece	690 (7–18 years)	C-S	KidMed^c^	Current wheezing, ever wheezing, wheezing ever with atopy	Not associated with current and ever wheezing	Age, sex, BMI, parental asthma, number of older siblings
					Allergic rhinitis ever, allergic rhinitis ever with atopy, current allergic rhinitis	adjOR = 0.34 [0.18–0.64], *p* < 0.001 for allergic rhinitis ever; adjOR = 0.39 [0.13–0.97], *p* < 0.05 for allergic rhinitis ever with atopy; adjOR = 0.49 [0.24–0.99], *p* < 0.05 for current allergic rhinitis	

Garcia-Marcos et al. (8)	Spain	20,106 (6–7 years)	C-S	Mod.Psal 2^a^	Current occasional asthma, current severe asthma	adjOR = 0.90 [0.82–0.98] for current severe asthma in girls	Older and younger sibling, maternal smoking
					Current rhinoconjunctivitis	adjOR = 0.98 [0.93–1.03] for current rhinitis in girls, adjOR = 0.99 [0.95–1.03] for current rhinitis in boys	

Chatzi et al. (10)	Spain	460 (6.5 years)	C-S	KidMed^c^	Persistent wheeze, atopic wheeze	Not associated with either asthma outcomes	Sex, maternal and paternal asthma, maternal social class and education, BMI, total energy intake

Castro-Rodriguez et al. (11)	Spain	1784 (4.1 ± 0.8 years)	C-S	Mod.Psal 1^b^	Current wheeze	adjOR = 0.54 [0.33–0.88]	Several factors^e^

Romeiu et al. (15)	Mexico	158 asthmatic (9.6 years)	Cohort	Mod.Trich^d^	Inflammatory response (IL-8), lung function (FEV1, FVC)	Positively associated with FEV1 (*p* = 0.045) and FCV (*p* = 0.018) and was modifier for the effect of ozone on FVC (*p* = 0.02)	Sex, BMI, previous day min. temperature, corticoid use, chronological time
		50 non-asthmatic (9.3 years)					

De Batlle et al. (12)	Mexico	1,476 (6–7 years)	C-S	Mod.Trich^d^	Ever asthma, ever wheezing, current wheezing	adjOR = 0.60 [0.40–0.91] for ever asthma; adjOR = 0.64 [0.47–0.87] for ever wheezing	Sex, maternal education, exercise, current ETS at home, maternal asthma and rhinitis
					Rhinitis ever, current rhinitis, current sneezing, current itchy-water eyes	adjOR = 0.64 [0.36–1.15] for rhinitis ever; adjOR = 0.87 [0.65–1.18] for current rhinitis; adjOR = 0.71 [0.53–0.97] for current sneezing; adjOR = 0.96 [0.64–1.45] for current itchy-watery eyes	

Nagel et al. (13)	20 countries	50,004 (8–12 years)	C-S	Mod.Psal 2^a^	Ever asthma, current wheeze, atopic wheeze, BHR	adjOR = 0.95 [0.94–0.99] for ever asthma; adjOR = 0.97 [0.94–0.99] for current wheezing; adjOR = 1.05 [0.96–1.14] for BHR	Age, sex, ETS, parental atopy, exercise, siblings
					SPT	No association with positive SPT (adjOR = 1.00 [0.95–1.04])	

Gonzalez-Barcala, et al. (16)	Spain	14,700 (6–7 and 13–14 years)	C-S	Mod.Psal 2^a^	Prevalence and severity ever asthma	adjOR = 2.26 [1.2–4.2] among girls aged 6–7 years	BMI, parental smoking, maternal education

Suarez-Varela, et al. (25)	Spain	20,106 (6–7 years)	C-S	Mod.Psal 2^a^	Atopic dermatitis	adjOR = 1.03 [0.99–1.08], *p* = 0.071	Gender, obesity, ETS first year of livelife, siblings, exercise

Arvaniti et al. (14)	Greece	700 (10–12 years)	C-S	KidMed^c^	Ever asthma, asthma symptoms, ever wheeze, exercise wheeze	Lower ever asthma, any asthma symptoms, ever wheeze, and exercise wheeze (all *p* < 0.005)	Age, sex, BMI, physical activity status, energy intake

Grigoropoulou et al. (17)	Greece	1,125 (10–12 years)	C-S	KidMed^c^	Ever asthma (symptoms)	adjOR: 0.84 [0.77–0.91]	Age, gender, BMI, physical activity status, and energy intake

Akcay et al. (18)	Turkey	9,991 (13–14 years)	C-S	Mod.Psal 2^a^	Asthma diagnosis by physician	No significant association	Not adjusted

Tamay et al. (26)	Turkey	10,984 (13–14 years)	C-S	Mod.Psal 2^a^	Physician-diagnosed allergic rhinitis	No difference on physician-diagnosed allergic rhinitis	Not adjusted

Tamay et al. (27)	Turkey	11,483 (6–7 years)	C-S	Mod.Psal 2^a^	Physician-diagnosed allergic rhinitis, current rhinoconjunctivitis, lifetime rhinitis, current rhinitis	OR = 0.97 [0.94–0.99], *p* = 0.007 for physician diagnosed allergic rhinitis, OR = 0.97 [0.94–0.99], *p* = 0.01 for current rhinoconjunctivitis, OR = 0.97 [0.96–0.98], *p* < 0.001 for lifetime rhinitis. But after being adjusting no association with any outcome was found	Exercise frequency, hours spent on watching TV/computer, foods

Alphantonogeorgos et al. (19)	Greece	1,125 (10–12 years)	C-S	KidMed^c^	Ever asthma (symptoms)	Low adherence associated with more asthma (rural area *p* = 0.04, urban area *p* < 0.001). Mediation through the MedDiet (beta = −0.029, *p* < 0.001) reduced the harmful effect of urban living	Age, gender, parental history of atopy

Silveira et al. (20)	Brazil	268 persistent and 126 intermittent asthmatic (3–12 years)	C-S	Mod.Psal 2^a^	Persistent (mild, moderate or severe) vs. intermittent asthma	No difference for persistent vs. intermittent asthma (57 vs. 52%, *p* = 0.40)	Not adjusted

Rice et al. (21)	Peru	287 asthmatic and 96 controls (9–19 years)	C-C	Mod.Psal 1^b^	Asthma diagnosis by physician, ACT, lung function	OR = 0.56 [0.36–0.90], *p* = 0.02; adjOR = 0.55 [0.33–0.92], *p* = 0.02, for asthma. No difference in ACT, nor FEV1. Interaction with maternal education (higher MedDiet score with more education)	BMI, sex, age, maternal education
					Allergic rhinitis, atopy (specific IgE)	No association with any allergy outcome was found	

*ACT, asthma control test; adjOR, adjusted odds ratio; BMI, body mass index; C-C, case control; C-S, cross-sectional; ETS, environmental tobacco smoke; FVC, forced vital capacity; FEV1, forced expiration volume in the first 1 s; Ig, immunoglobulin; IL, interleukin; MedDiet, Mediterranean diet; OR, odds ratio; SPT, skin prick test*.

*^a^Modified from Psaltopoulou (23) (min–max score: 0–20)*.

*^b^Modified from Psaltopoulou (23) (min–max score: 0–36)*.

*^c^Modified from KIDMED (24)*.

*^d^Adapted from Trichopoulou (25)*.

*^e^Age, birth weight, livestock during pregnancy, cesarean, antibiotic and acetaminophen consumption during the previous 12 months, rhinoconjunctivitis, dermatitis, paternal and maternal asthma, maternal age and education level, current paternal and maternal smoking, vigorous physical activity, and cats at home in the last 12 months*.

However, seven studies, most of them recently published, were not included in the aforementioned review (6). Two studies were performed in Greece (17, 19), and one in each of the following countries: Mexico (15), Spain (16), Turkey (18), Brazil (20), and Peru (21). In three studies, adherence to MedDiet was significantly associated with lower asthma symptoms (17, 19, 21). Additionally, adherence to MedDiet was significantly associated with improved lung function in one study (15), but not in another one (21). By contrast, one study (16) showed higher asthma prevalence among girls with higher adherence to MedDiet. In two studies (18, 20), no significant effect of MedDiet was found. Interestingly, an inverse mediating effect of MedDiet was observed for the urban environment–asthma relation (standardized beta = −0.029, *p* < 0.001), while physical activity had no significant contribution, adjusted for several confounders (19). A direct interaction of MedDiet with maternal education was found in one of the studies (21).

### Effect of MedDiet Consumption by Children on Allergic Rhinitis and Dermatitis/Eczema

With respect to the association between MedDiet consumption and allergic rhinitis and/or atopic dermatitis, 8 studies were retrieved (Table 1). Two studies were carried out in each of Spain (8, 25) and Turkey (26, 27), one was worldwide (13), and one in each of the following countries: Greece (9), Peru (21), and Mexico (12). High adherence to MedDiet was significantly protective for allergic rhinitis and atopy in only one study (9) as measured by skin prick test (SPT). However, no effect on allergic rhinitis was demonstrated in the other four studies (8, 21, 26, 27). Moreover, no significant effect of MedDiet was demonstrated on atopic dermatitis (25), nor on atopic sensitization as defined either by specific IgE (21) or by SPT (13).

### Effect of MedDiet Consumption by Mother on Asthma/Wheeze/Allergic Diseases in the Offspring

Seven studies that explore this effect were retrieved (Table 2). Four studies were done in Spain (10, 28–30), one in Mexico (12), one in the US (31), and one in Greece/Spain (32). In all of them, the outcome was asthma/wheeze. In three studies, atopic eczema/atopy was included (30–32), and in two studies, rhinitis was also included (12, 30). The offspring were surveyed at different times. In three studies, it was during the first year of life (28, 29, 32), at 3 years in one study (31), at 4 years in another one (30), and at 6.5 years of life in the others (10, 12).

**Table 2:** **Summary of studies reporting the association between adherence to MedDiet during pregnancy and asthma/wheezing/allergic disease in offspring**.

Reference	Country	Sample of (mother/offspring)	Study design	MedDiet score	Asthma, dermatitis, and allergic rhinitis outcomes	Primary results: effect of MedDiet	Adjusted confounders
Chatzi et al. (10)	Spain	460	C-S	KidMed^b^	Persistent wheeze and atopic (SPT) wheeze at 6.5 years	A high MedDiet Score during pregnancy was protective for persistent wheeze (adjOR = 0.22 [0.08–0.58]), atopic wheeze (adjOR = 0.30 [0.10–0.90]), and atopy (adjOR = 0.55 [0.31–0.97]) at age 6.5 years	Sex, maternal and paternal asthma, maternal social class and education, BMI, and total energy intake at age 6.5 years
De Batlle et al. (12)	Mexico	1,476	C-S	Mod.Trich^c^	Ever asthma, ever wheezing, current wheezing, rhinitis ever, current rhinitis, current sneezing, and current itchy-watery eyes at 6–7 years	No associations were found between mothers diet score during pregnancy and asthma or allergic rhinitis outcomes in children in the crude or adjusted analyses, except for current sneezing = 0.71 [0.53–0.97]	Sex, maternal education, exercise, current tobacco smoking at home, maternal asthma and maternal rhinitis
Castro-Rodriguez et al. (28)	Spain	1,409	Cohort	Mod.Psal 1^a^	Any wheeze at first year	MedDiet (*p* = 0.036) and olive oil (*p* = 0.002) during pregnancy were significantly associated with less wheezing. Only olive oil consumption remained associated (adjOR = 0.57 [0.4–0.8], *p* = 0.002)	Sex, exclusive breastfeeding, day care, eczema, maternal asthma, smoking during pregnancy, siblings, mold stains on household wall, and preterm birth
Lange et al. (31)	US	1,376	Cohort	Mod.Trich^c^	Recurrent wheeze, doctor diagnosis of asthma and atopy at 3 years	OR = 0.64 [0.43–0.95], adjOR = 0.98 [0.89–1.08] for recurrent wheeze. No association with doctor’s diagnosis of asthma, or atopy	Sex, maternal race, maternal education level, household income, maternal and paternal history of asthma, presence of children at home, maternal pre-pregnancy, BMI, breast feeding duration, passive smoke exposure
Chatzi et al. (32)	Spain and Greece	Spain: 1,771; Greece: 745	Cohort	Mod.Trich^c^	Wheeze and eczema at 12 months	Not associated with risk of wheeze and eczema	Not adjusted
Pellegrini-Belinchón et al. (29)	Spain	1,164	Cohort	None	Recurrent wheeze at first year	adjOR = 0.436 [0.297–0.640]	Nursery, eczema, maternal asthma, smoking in third trimester
Castro-Rodriguez et al. (30)	Spain	1,001	Cohort	Mod.Psal 1^a^	Current wheeze, dermatitis and allergic rhinitis at 4 years	MedDiet score adherence by mother and by child at year 4 did not remain a protective factor for any outcome	Many environment factors^d^

*adjOR, adjusted odds ratio; BMI, body mass index; C-S, cross-sectional; MedDiet, Mediterranean diet; OR, odds ratio; SPT, skin prick test*.

*^a^Modified from Psaltopoulou (22) (min–max score: 0–36)*.

*^b^Modified from KIDMED (23)*.

*^c^Adapted from Trichopoulou (24)*.

*^d^Age, birth weight and height, cesarean, antibiotic and acetaminophen consumption during the previous 12 months, oral contraception use, parental asthma, parental rhinitis, parental dermatitis, maternal age and education level, current paternal and maternal smoking, breastfeeding, siblings, pets at home during pregnancy, mold stain, day care, type of fuel, TV video and physical activity at 4 years, air pollution, and colds during first year of life*.

Adherence to MedDiet by the mother had a protective effect on wheeze during the first year of life in the offspring in one study (29), and this effect disappeared after adjusting for confounders in another study (28). In three studies (30–32), the effect of maternal MedDiet had no significant effect on wheeze. When combined maternal and child adherence to MedDiet was analyzed, only one study showed a protective effect on persistent and atopic wheezing, and atopy by SPT at 6.5 years of age (10). However, another study did not find any such association (30). Regarding atopic eczema and allergic rhinitis, adherence to MedDiet by the mother had no significant protective effect in the majority of the studies, except for one (12) in which maternal MedDiet consumption was protective for current sneeze in the offspring at 6–7 years of age.

In summary, adherence to MedDiet by children themselves seems to have a protective effect on asthma/wheezing symptoms after adjusting for confounders, but the effect is doubtful on lung function and bronchial hyperresponsiveness. By contrast, the vast majority of the studies showed no significant effect of MedDiet on preventing allergic diseases (atopic eczema, rhinitis, or atopy). Finally, studies on adherence to MedDiet by the mother during pregnancy showed some protective effect on asthma/wheeze symptoms in the offspring only during their first year of life, but not after. Only very few studies showed a protective effect on wheezing, current sneeze, and atopy. No study has shown any protective effect on atopic dermatitis.

The results of the protective effect of MedDiet on asthma/wheezing symptoms in children found in the present review expand the results reported in systematic reviews with meta-analyses previously carried out by our group (6) and by others (33). The present report also confirms the protective effect of MedDiet consumption by the mother on asthma or allergic diseases in their offspring, as was described in a different review published by our group (34) and by others (35, 36), suggesting some epigenetic effect of diet. However, no systematic review on the effect of MedDiet on other allergic diseases, i.e., rhinitis or atopic eczema has been previously published. In the present study, no effect was shown in seven out of eight studies.

## How MedDiet Might Work?

In the past decades, one of the most important environmental changes worldwide has been that of diet patterns. Due to changes in dietary fat intake (by increasing *n* − 6 PUFAs and decreasing *n* − 3 PUFAs), through an increased production of prostaglandin (PG) E2 might have contributed to the increase in the prevalence of asthma (37). PGE2 suppresses T-helper (Th) 1 and increases Th2 phenotype, thus reducing IFN-gamma. Th2 phenotype, in which there is an increase of IgE isotype switching, is associated with asthma and atopic diseases. On the other hand, diets low in antioxidants may have increased the susceptibility to develop asthma (38). In this case, the proposed mechanism involves the decline of lung antioxidant defenses, resulting in increased oxidant-induced airway damage.

Several of those studies reported on the beneficial effect of single foods rich in *n* − 3 PUFA or antioxidants on asthma or allergic diseases. A meta-analysis concluded that increased consumption of vegetables and fruits, zinc, and vitamins A, D, and E, is related to lower prevalence of asthma and wheeze in children and adolescents (36). However, most of the studies included in that meta-analysis fail to account for the interactions between nutrients (39). Yet, since our normal eating behavior is to follow certain diet patterns that contain many specific foods, it seems reasonable to put the focus on food patterns or diets.

Mediterranean diet is rich in both antioxidants and *cis*-monounsaturated fatty acids. Moreover, cereals (whole grain) are rich in vitamin D, phenolic acids, and phytic acid. Moreover, fruits, vegetables, and legumes are rich in vitamins C and E, carotenoids, selenium, and flavonoids (5). Additionally, olive oil for cooking and dressing salads is an important part of MedDiet. The main active components of olive oil are oleic acid, phenolic derivatives (hydroxytyrosol, tyrosol, oleuropein, and ligstroside), and squalene, all of which have been found to exhibit a marked antioxidant activity. Also, oleuropein and hydroxytyrosol (its hydrolysis product) are among the most potent antioxidants (40). One study has shown that, after a multivariate analysis, olive oil consumption by the mother during pregnancy, but not MedDiet, remained as a protective factor (aOR = 0.57 [0.4–0.9]) for wheezing during the first year of life in the offspring (28). Therefore, it seems reasonable to think that a higher adherence to MedDiet or olive oil may have some protective effects on asthma and allergic diseases in childhood. The ability of this diet to counteract oxidative stress might have an effect on asthma inception (41).

## What is Needed?

All studies included in the present review were observational (cross-sectional, cohort, and case–control) studies. All but one of them used three different MedDiet scores: one modified from Psaltopoulou (22), one modified from KIDMED (23), and another one adapted from Trichopoulou (24). Seventeen studies come from Mediterranean countries (nine from Spain, five from Greece, and three from Turkey). While these observational studies have reported potentially beneficial associations with MedDiet on asthma/wheezing, it is unknown whether an intervention to promote the MedDiet could reduce the prevalence of asthma and allergic diseases in children.

Currently, there are no RCTs testing the hypothesis that adherence to MedDiet could decrease the risk of asthma and allergic disease in children. One pilot RCT in pregnant Scottish women at high risk of having asthmatic or allergic offspring is ongoing (42). It aims are to establish recruitment, retention, and acceptability of the dietary intervention and to assess the likely impact of the intervention on adherence to a MedDiet during pregnancy through seeking a reduction in the incidence of asthma and allergic problems (42). In adults, only one 12-week open-label trial was published (43). In that small study, 38 asthmatic adults were randomized to receive either 41 h of dietician services or only 2 h of services, or just one dietician session and recipes (control). The study achieved its primary outcome of altering the eating habits of participants in the high-intensity intervention toward a MedDiet pattern. The study had not enough power to detect clinical endpoints; however, non-significant improvements in asthma-related quality of life, asthma control, or spirometry were observed in the intervention groups (43).

## Conclusion

Adherence to MedDiet by children seems to have a protective effect on asthma/wheezing symptoms, but not on allergic rhinitis, eczema, or atopy. Adherence to MedDiet by the mother during pregnancy might have some protective effect on asthma/wheeze symptoms in the offspring only during their first year of life, and few studies have shown some protective effect on current sneeze and atopy, but none on eczema. Randomized control trials on MedDiet adherence to test the real usefulness on primary prevention (by maternal consumption during pregnancy) or on clinical improvement (by patient consumption) of asthma/wheezing, allergic rhinitis, and dermatitis in childhood are needed.

## Author Contributions

JC-R contributed to the study concept, literature search, data collection, and manuscript writing. LG-M contributed to the literature search, data collection, and manuscript writing.

## Conflict of Interest Statement

The authors declare that the research was conducted in the absence of any commercial or financial relationships that could be construed as a potential conflict of interest.
